# Phyto-SERM Constitutes from *Flemingia macrophylla*

**DOI:** 10.3390/ijms140815578

**Published:** 2013-07-26

**Authors:** Wan-Chun Lai, Ya-Ting Tsui, Abdel Nasser B. Singab, Mohamed El-Shazly, Ying-Chi Du, Tsong-Long Hwang, Chin-Chung Wu, Ming-Hong Yen, Ching-Kuo Lee, Ming-Feng Hou, Yang-Chang Wu, Fang-Rong Chang

**Affiliations:** 1 Graduate Institute of Natural Products, College of Pharmacy, Kaohsiung Medical University, Kaohsiung 80708, Taiwan; E-Mails: stellapple7@gmail.com (W.-C.L.); shinygirl1983@hotmail.com (Y.-T.T.); ycdu0626@gmail.com (Y.-C.D.); ccwu@kmu.edu.tw (C.-C.W.); yen@kmu.edu.tw (M.-H.Y.); 2 Department of Pharmacognosy and Natural Products Chemistry, Faculty of Pharmacy, Ain-Shams University, Organization of African Unity Street, Abassia, Cairo 11566, Egypt; E-Mails: dean@pharma.asu.edu.eg (A.N.B.S.); elshazly444@googlemail.com (M.E.-S.); 3 Graduate Institute of Natural Products, Chang Gung University, Taoyuan 333, Taiwan; E-Mail: htl@mail.cgu.edu.tw; 4 Graduate Institute of Pharmacognosy, Taipei Medical University, Taipei 110, Taiwan; E-Mail: cklee@tmu.edu.tw; 5 Cancer Center, Kaohsiung Medical University Hospital, Kaohsiung 80708, Taiwan; E-Mail: mifeho@kmu.edu.tw; 6 School of Pharmacy, College of Pharmacy, China Medical University, Taichung 404, Taiwan; 7 Chinese Medicine Research and Development Center, China Medical University Hospital, Taichung 404, Taiwan; 8 Center for Molecular Medicine, China Medical University Hospital, Taichung 404, Taiwan

**Keywords:** *Flemingia macrophylla*, menopausal, phytoestrogen, fleminigin, flemichin E, pER8:GUS

## Abstract

The methanolic extract of *Flemingia macrophylla* roots exhibited significant estrogenic activity in the transgenic plant assay system which was comparable to the activity of soybean extract. Utilizing estrogenic activity-guided fractionation, one new compound, fleminigin, together with 23 known compounds were isolated from *F. macrophylla* roots’ methanolic extract. The structure of the new compound was identified based on intensive spectroscopic analysis and the full spectral data for one of the isolated compounds, flemichin E, was introduced for the first time in the current investigation. The estrogenic and anti-estrogenic activities of the isolated compounds were evaluated revealing that the isolated isoflavonoids may act as partial estrogen agonists, as well as antagonists. Additionally, the anti-inflammatory and the cytotoxic activities of the isolated compounds were studied. These results suggested the potential applications of *F. macrophylla* extract and its isolated compounds as selective estrogen receptor modulators (SERMs).

## 1. Introduction

Plants with estrogenic properties have been widely used as dietary supplements for postmenopausal women in the last few decades [1]. They possess a myriad of protective physiological activities against neurological diseases [2], hypertension [3], cardiovascular diseases [4] and excessive oxidative reactions [5,6]. Identification of phytoestrogenic natural sources from thousands of terrestrial plants is a challenging task, which represents the bottle neck in developing new dietary supplements and pharmaceutical lead drugs. This stern challenge was tackled by several research groups in the past few decades developing *in vitro* techniques for their detection. One of the highly efficient detection techniques is the transgenic plant system, pER8:GUS, which contains a human estrogen receptor (ER) and β-glucuronidase (GUS) reporter genes [7]. The transgenic plant system can be easily utilized to screen the estrogenic activity of extracts and pure compounds with high efficiency and versatility [8]. In our ongoing research to discover new sources of phytoestrogens, 22 herbal (folk) medicines used in the treatment of fractures, osteoporosis, and related bone diseases were selected for evaluating their estrogenic activity. Among these herbs, the methanolic (MeOH) extract of *Flemingia macrophylla* (Willd.) Kuntze ex Prain roots showed significant estrogen–like activity with a minimum active concentration (MAC) of 0.78 μg/mL, which was higher but still comparable to soybean extract, a standard phytoestrogenic extract with a MAC of 0.25 μg/mL.

*F. macrophylla* is a shrubby herb which is widely distributed in southern China, India, Indonesia, and Taiwan [9]. The roots of this plant have been used in folk medicine for the treatment of fractures, trauma, arthritis, rheumatism, and influenza. Previous studies reported that *F. macrophylla* crude extract exhibited neuroprotective [10], analgesic [11], and anti–inflammatory activities [11]. It was also found to be effective in the treatment of osteoporosis [12]. Recent reports have suggested a close relationship between *F. macrophylla* estrogen-like activity and its neuroprotective [10] and anti-osteoporotic activities [12]. Despite these findings, the effect of *F. macrophylla* extracts and its purified compounds on estrogen receptors has never been investigated.

In this study, the estrogenic/antiestrogenic effects of *F. macrophylla* extract and its constituents were investigated using a transgenic plant assay system as well as an *in vitro* reporter assay in human breast cancer MCF-7 cell line. Bioactivity-guided fractionation of the extract led to the isolation of 24 compounds. Moreover, the anti–inflammatory and cytotoxic activities of the isolated compounds were also evaluated.

## 2. Results and Discussion

### 2.1. Structure Elucidation

**Figure 1:**
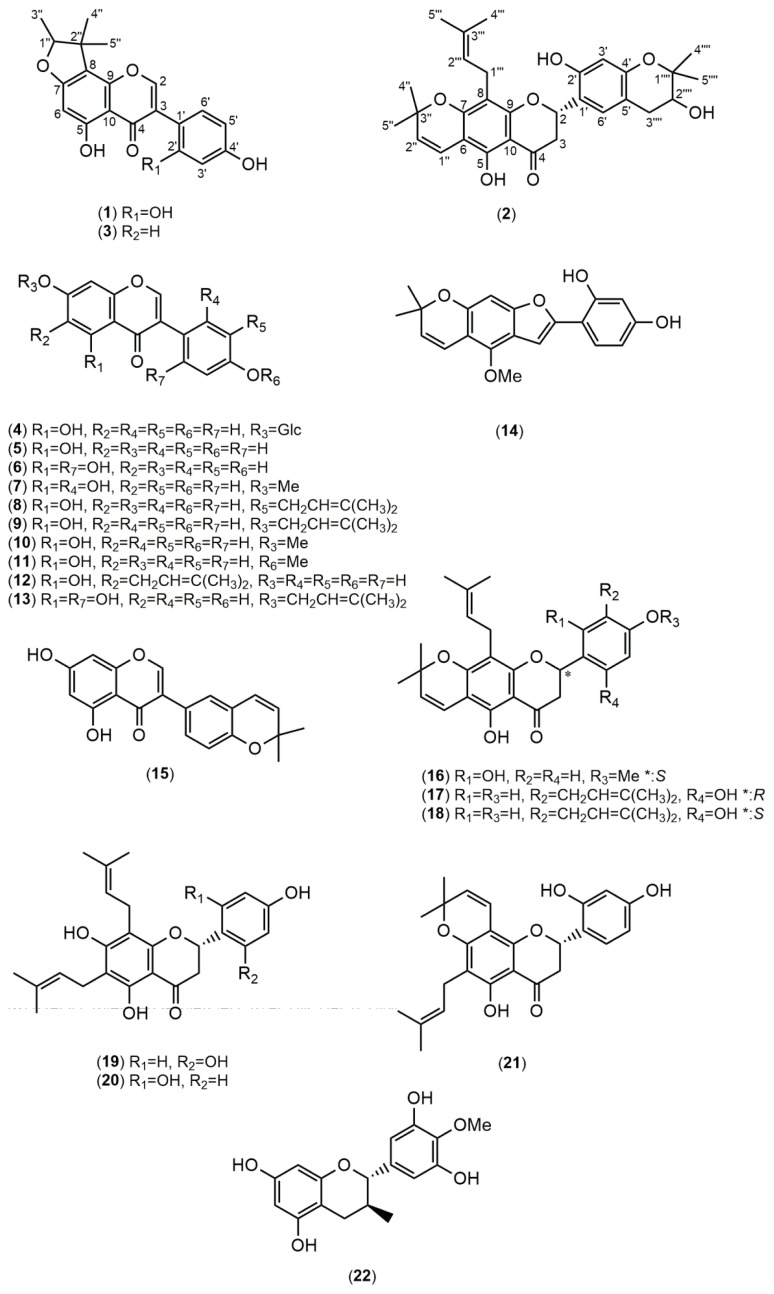
Structures of the isolated compounds from *F. macrophylla.*

**Figure 2:**
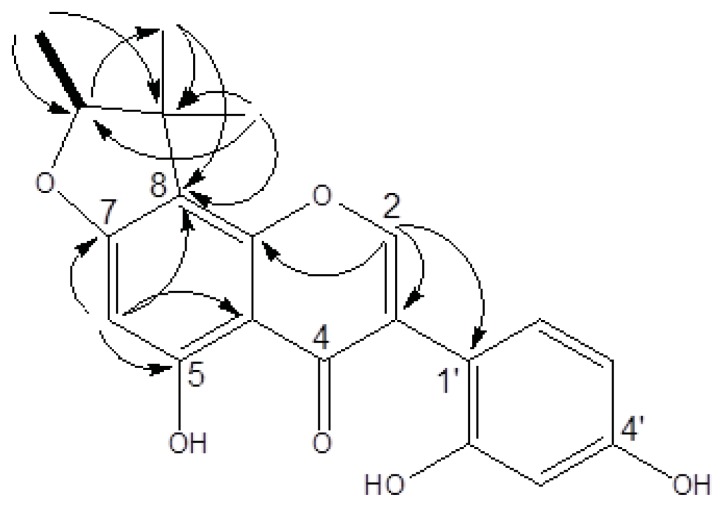
^1^H-^1^H COSY (correlation spectroscopy) and HMBC (heteronuclear multiple bond correlation) correlations of fleminigin (**1**).

Using bioactivity-guided fractionation, the MeOH extract of *F. macrophylla* roots was fractionated by column chromatography over silica gel and purified by HPLC. Purification of the extract led to the isolation of one new compound, fleminigin (**1**) and 23 known compounds, flemichin E (**2**) [13], flemiphilippinin F (**3**) [14], genistin (**4**) [15], genistein (**5**) [16], 2′-hydroxygenistein (**6**) [17], cajanin (**7**) [18], 3′-isoprenylgenistein (**8**) [19], 7-(3,3-dimethylallyl)genistein (**9**) [20], prunetin (**10**) [21], olmelin (**11**) [22], erythrinin B (**12**) [23], 5,2′,4′-trihydroxy-7-(3-methylbut-2-enyloxy)isoflavone (**13**) [24], neoraufurane (**14**) [25], isoderrone (**15**) [17], fleminone (**16**) [26], an enantiomer of 5,2′,4′-trihydroxy-8,5′-di-(3-methylbut-2-enyl)-6,7-3,3-dimethylpyrano)flavanone (**17** and **18**) [20], flemiphilippinin D (**19**) [27,28], flemiflavanone A (**20**) [29], flemichin-D (**21**) [27], 4′-*O*-methylgallocatechin (**22**) [30], β-sitosterol (**23**) [31], and stigmasterol (**24**) [31] were obtained (Figure 1). Among the known compounds, compound **2** was identified in a previous report using mass spectrometry, but its complete 1D and 2D NMR data have never been reported. Compounds **7**–**10** and **13**–**15** were isolated from this species for the first time. Additionally, compound **9** was isolated for the first time from natural sources (Figure 2).

Compound **1** was obtained as a yellow amorphous solid. The HRESIMS of **1** showed a molecular ion at *m*/*z* 377.1001 [M + Na]^+^, indicating a molecular formula of C_20_H_18_O_6_ (calculated 377.1005). UV absorptions at 263, 295, and 335 nm suggested the presence of an isoflavone skeleton. The IR spectrum showed characteristic absorptions for a hydroxy (3377 cm^−1^), carbonyl (1651 cm^−1^), and aromatic (1615 and 1506 cm^−1^) functionalities. The ^1^H NMR of **1** (in acetone-*d*_6_) showed a signal at δ_H_ 8.19 (1H, s), which represented a characteristic signal of H–2 in the isoflavone nucleus (Table 1). From the COSY spectrum, the correlation of one oxymethine (δ_H_ 4.57, 1H, q, *J* = 6.4) with one methyl (δ_H_ 1.4, 3H, d, *J* = 6.4), as well as the observed HMBC correlations (Figure 2), suggested that a furan moiety is fused to ring A in **1**. The ^1^H and ^13^C NMR data of **1** (acetone-*d*_6_, Table 1) were similar to those of a previously identified isoflavone, known as 5,7,4′-trihydroxy-4″,4″,5″(ξ)-trimethyl-4″,5″-dihydro-furano-(7,6,2″,3″)isoflavone [32]. Guided by the reported data of the known compound, we were able to elucidate the structure of compound **1**. Acetone-*d*_6_ and pyridine-*d*_5_ were used as solvents for ^1^H and ^13^C NMR analysis of **1** and the spectra were compared to those of the known compound [32]. For example, in compound **1** we noticed an ABX spin systems at δ_H_ 6.49 (1H, d, *J* = 2.2), 6.43 (1H, dd, *J* = 8.4, 2.2), and 7.12 (1H, d, *J* = 8.4) indicating the presence of unusual 1′,2′,4′-trisubstitutions on ring B as well as an aromatic proton on ring A at δ_H_ 6.22 (1H, s). These data were similar to those reported for the known compound [32].

The HMBC spectrum of the known compound showed correlations between the singlet aromatic proton (H-8, δ_H_ 6.54) and the two carbons attached to the oxygen atom in ring A (C-7 and C-9) (δ_C_ 153.9 and 164.4) [32] indicating that the furan moiety is fused to C-6 and C-7 of ring A. On the other hand, the HMBC spectrum of **1** showed that the singlet aromatic proton (H-6, δ_H_ 6.22) is correlated to two different carbons in ring A (C-5 and C-7) (δ_C_ 166.7 and 164.8), suggesting that the furan moiety is linked to C-7 and C-8 of ring A (Figure 2). Based on the aforementioned data, the structure of the new compound **1** was identified as 5,2′,4′-trihydroxy-7,8-(1,2,2-trimethyl-dihydrofurano) isoflavone, and was named fleminigin (**1**).

Compound **2** was obtained as a yellow amorphous solid. The HRESIMS of **2** showed a molecular ion peak at *m/z* 529.2202 [M + Na]^+^, corresponding to the molecular formula C_30_H_34_O_7_ (calculated 529.2199). Full ^1^H and ^13^C NMR data are summarized in Table 2 as well as the 2D NMR spectra, which were introduced for this compound for the first time. The planar structure of **2** was confirmed by ^1^H and ^13^C NMR data which were compared to the incomplete NMR data previously reported for this compound [13]. In the NOESY spectrum, H–2‴′ (δ_H_ 3.80, 1H, dd, *J* = 5.4, 4.8) showed a correlation with H-5‴′ (1.33, s), confirming the relative configuration of H-2‴′ and the methyl group (H-5‴′) as *cis*. However, the absolute configuration of C-2‴′ is still uncertain. By comparing the CD values of **2** with the reported data of similar structures [28], a negative cotton effect at 300 nm and a positive cotton effect at 340 nm indicated that the stereo configuration of C-2 is *S*. Thus, compound **2** was identified as (2*S*)-5,2′-dihydroxy-6,7-(1,1-dimethylpyrano)-8-(3-methylbut-2-enyl)-4′,5′-(2-hydroxy- 1,1-dimethylpyrano)flavanone, or flemichin E (**2**).

### 2.2. Estrogenic and Anti-Estrogenic Activities

The isolated compounds were subjected to the estrogen receptor (ER) binding affinity evaluation assay utilizing an efficient transgenic plant assay system [8]. The results showed that compounds **4**, **6**, **7**, and the reference drug, genistein (**5**), exhibited a significant estrogenic activity (MAC < 5 μM) in the transgenic plant assay system (Table 3). Isoflavone derivatives, compounds **1**, **3**, **8**, **12**, and **13**, showed a moderate estrogenic activity. However, the flavone derivatives, compounds **19** and **21**, exhibited weak estrogenic activity only at high doses (MAC > 50 μM). Compound **22** even showed weaker estrogenic activity exclusively at higher concentration (MAC > 300 μM).

**Figure 3:**
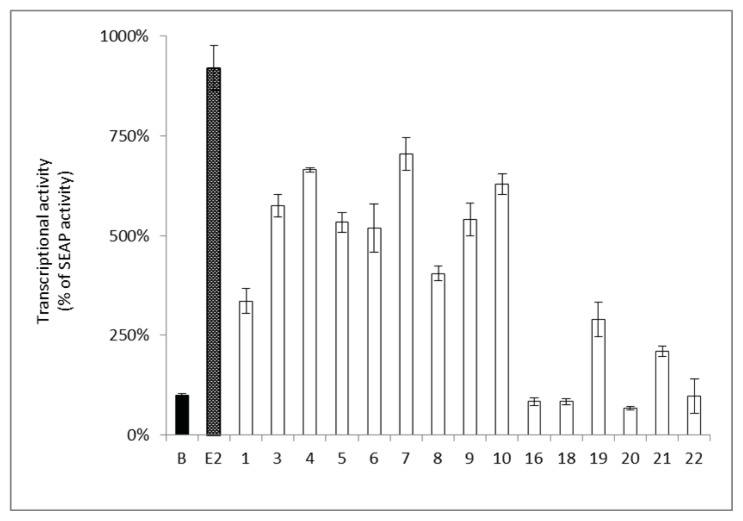
Estrogenic activity in MCF-7 cells of pure components from *F. macrophylla.* The SEAP activity induced by the positive control, E2 (0.1 nM). Cells were treated by the target compounds (10 μM) and the SEAP activity was compared to B (Blank), which was set to 100% SEAP activity. Each column represents a percentage of SEAP activity and is expressed as mean ± SEM (*n* = 4).

Furthermore, the estrogenic activity of the isolated compounds was confirmed by the transcription of an estrogen responsive element (ERE) utilizing human breast cancer ER–positive MCF-7 cells. The activity of the isolated compounds on the ERE was determined by the detection of the secreted alkaline phosphatase (SEAP) reporter protein. The results showed that compounds **3**, **4**, **6**, **7**, **9**, and **10** significantly increased the estrogenic activity (increase in SEAP% by 500%–700%) at a concentration of 10 μM (Figure 3), which was comparable to those of genistein (**5**) at 10 μM (Figure 3). Compounds **1**, **8**, **19**, and **21** showed a slight increase in the estrogenic activity (increase in SEAP% < 500%) compared to the basal activity (SEAP% 100%). However, compounds **16**, **18**, **20**, and **22** did not show any estrogenic activity (Figure 3).

**Figure 4:**
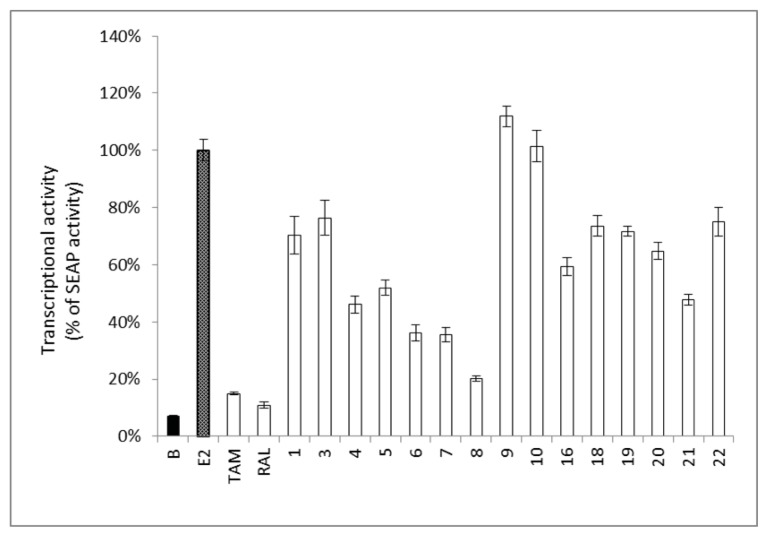
Antiestrogenic activity in MCF-7 cells of pure components from *F. macrophylla*. The SEAP activity induced by E2 (0.1 nM) was set to 100%. Cells treated with E2 (0.1 nM) and tamoxifen (TAM, 2 μM) or raloxifene (RAL, 20 nM), were selected as positive controls. Pure compounds (10 μM) were treated in the presence of E2 (0.1 nM). Each column represents a percentage of SEAP activity and is expressed as mean ± SEM (*n* = 4).

Certain phytoestrogens and estrogenic active compounds may possess counter pharmacological activity through acting as antiestrogenic agents, and they are known as selective estrogen receptor modulators (SERMs) [33]. Therefore, the isolated compounds were treated with E2 (0.1 nM) to determine their antiestrogenic activity in the ER–positive MCF-7 cell line. Results showed that compounds **4**–**8** reduced the ERE transcriptional activity of MCF-7 cells in the presence of E2 (Figure 4), which suggests that **4** and **6**–**8** may act as partial estrogen antagonists similar to genistein (**5**) [34]. Interestingly, 3′-isoprenylgenistein (**8**) showed a more potent anti-estrogenic effect compared to genistein (**5**). This dual activity opens a new avenue for selecting and designing a perfect SERM, acting preferentially on the nervous and cardiovascular systems, with minimum activity on mammary glands and uterus.

Several research groups have shown that isoflavonoids exhibited more potent estrogenic activity compared to flavonoids [35]. Certain structural features in isoflavonoids seemed to affect their phytoestrogenic activity. The parent skeleton should have at least a single hydroxy group in ring A or B. The highest estrogenic activity resulted from the presence of two hydroxy groups at the longitudinal extremities of the skeleton at C-7 and C-4′ [36]. Through studying the estrogenic activity of the isolated compounds, we found that the most active compound was genistein (**5**) with two hydroxy groups at C-7 and C-4′. It has an additional hydroxy group at C-5 which forms an intramolecular hydrogen bond to the carbonyl group improving the hydrogen bond formation ability of 7-OH to the ER binding site [37,38].

On the other hand, the presence of 2′-OH as in **6** resulted in an 11–fold reduction of the estrogenic activity (Table 3). The activity was even more dramatically reduced when 7-OH was replaced by *O*-isoprenyl substitution as in **13**. Glucosylation of the hydroxy group at C-7 as in **4** resulted in a 3–4 fold decrease in activity compared to genistein (**5**) implying the importance of a free hydroxy group at C-7 [37]. This observation was also supported by the marked decrease in the estrogenic activity (60 folds) of compound **7** possessing a methoxy group at C-7 (Table 3). Compound **12** showed only weak estrogenic activity suggesting that the presence of 6-isoprenyl moiety reduced the ER binding affinity. Moreover, the presence of 3′-isoprenyl unit (**8**) or 7,8-furan ring (**1** and **3**) reduced the estrogenic activity (MAC 35.31–37.98 μM) (Table 3). The estrogenic activity of the prenylated flavonoids (**16**, **19**, and **21**) was lower compared to isoflavonoids. However, the data of the *in vitro* reporter assay (Figure 4) revealed that compounds **3**–**7** exhibited comparable estrogenic activity. The data collected from both assays suggested that the estrogenic activity of the isoflavonoids may be attributed to the ER-dependent or ER-independent pathways [39].

### 2.3. Anti–Inflammatory and Cytotoxic Activities

The anti-inflammatory activity of *F. macrophylla* crude extract has been recently reported [11]. The authors utilized λ-carrageenan to induce inflammation and paw edema in mice. The inflammation response has been linked to the neutrophils’ infiltration and the production of neutrophil-derived free radicals [11]. There is considerable evidence from clinical and experimental studies that the recruitment of neutrophils into joints is a critical hallmark of rheumatoid arthritis [40,41].

The anti-inflammatory activity was measured through testing the inhibition of superoxide anion generation and elastase release by neutrophils after induction with formyl-methionyl-leucyl-phenylalanine (fMLP) and cytochalasin B (CB). Compounds **3**, **6**, **7**, and **8** showed antioxidant activity against the production of superoxide anions (Table 4). Compounds **6** and **7** were able to compete in the inhibition of elastase release (IC_50_ 4.32 and 4.33 μg/mL) to the same level demonstrated by the positive control, genistein (**5**) (IC_50_ 4.25 μg/mL). A recent report [42] has suggested the therapeutic potential of genistein in targeting rheumatoid arthritis through its anti-inflammatory effect. The structural similarity between compounds **6**, **7**, as well as **8** and genistein (**5**) suggests a similar anti-inflammatory effect for these compounds which should be confirmed with further *in vitro* and *in vivo* studies.

We also evaluated the cytotoxic activity of the isolated compounds against different cancer lines. In the MTT cytotoxicity assay, the crude extract was inactive at 20 μg/mL. The tested compounds showed insignificant cytotoxicity and only compounds **19** and **21** exhibited marginal cytotoxicity against liver, breast, lung, and oral cancer cell lines.

## 3. Experimental Section

### 3.1. General Experimental Procedures

The used HPLC was composed of dual Shimadzu LC-10AT pumps and a Shimadzu SPD-10A UV-vis detector (Shimadzu Inc., Kyoto, Japan), as well as a Waters Atlantis T3 RP 150 × 4.6 mm, 5 μm preparative column (Waters Corp., Milford, CT, USA) or a Thermo ODS Hypersil 250 × 4.6 mm, 5 μm preparative column (Thermo Fisher Scientific Inc., Rockford, IL, USA). UV spectra were obtained using a JASCO UV-530 ultraviolet spectrophotometer (JASCO Inc., Tokyo, Japan). IR spectra were obtained on a Mattson Genesis II infrared spectrophotometer (Thermo Fisher Scientific Inc., Tokyo, Japan). Optical rotations were measured with JASCO DIP 370 and P-1020 digital polarimeters (JASCO Inc., Tokyo, Japan). NMR (400 MHz and 600 MHz) spectra were obtained on a Varian Unity 400 MHz FT-NMR and a Varian Unity 600 MHz FT-NMR (Varian Inc., Palo Alto, CA, USA). ESI-MS data were collected on a VG Biotech Quattro 5022 mass spectrometer (VG Biotech, Altrincham, UK). High-resolution ESI-MS data were obtained on a Bruker APEX II spectrometer (FT-ICR/MS, FTMS) (Bruker Daltonics Inc., Billerica, MA, USA).

Column chromatography (CC) was performed on silica gel (40–63 and 63–200 μm, Merck KGaA, Darmstadt, Germany). Preparative TLC and TLC analyses were performed on silica gel 60 F254 plates (Merck KGaA) and spots were visualized by UV and by spraying 50% H_2_SO_4_-ethanol solution followed by heating for 5 min.

Eagle’s phenol-red free minimum essential medium, fetal bovine serum, l-glutamine, and the antibiotic mixture (penicillin-streptomycin) were purchased from Invitrogen Co. (Invitrogen Co., Carlsbad, CA, USA). 3-(4,5-Dimethylthiazol-2-yl)-2,5-diphenyltetrazolium bromide (MTT), Na_3_PO_4_, EDTA, X-Gluc, and Triton X-100, and doxorubicin [(D1515-98.0%–102.0% (HPLC)] were purchased from Sigma (Sigma Chemical Co., St. Louis, MO, USA). Dimethyl sulfoxide (DMSO), K_3_Fe(CN)_6_, K_4_Fe(CN)_6_, were purchased from Merck (Merck KGaA). The purity of the isolated compounds used for biological assays was determined by HPLC (>95%). 17β-Estradiol (E2) was purchased from TCI (Tokyo Chemical Industry Co. Ltd., Tokyo, Japan).

### 3.2. Plant Material

The dried roots of *Flemingia macrophylla* (Willd.) Kuntze ex Prain (Flemingia-01) were collected from Taichung City, Taiwan, in February 2008. The material was identified by Dr. Ming-Hong Yen and deposited in the Graduate Institute of Natural Products, Kaohsiung, Taiwan.

### 3.3. Extraction and Isolation

The dried roots of *F. macrophylla* (7.7 kg) were extracted with 95% aqueous MeOH (10 L × 5) at room temperature and then concentrated under reduced pressure. The crude extract (654.0 g) was partitioned with H_2_O (2 L × 3) and EtOAc (2 L × 3) to yield a H_2_O layer (524.0 g), an insoluble portion (1.7 g) and an EtOAc layer (127.4 g). The insoluble portion was recrystallized from MeOH to yield genistin (**4**, 1.2 g). The EtOAc layer was then partitioned between n-hexane (2 L) and 90% aqueous MeOH solution (2 L × 3) to provide an n-hexane layer (35.5 g) and a MeOH layer (91.9 g). The MeOH layer exhibited the most potent estrogenic activity (Figure S1) and was selected for further bioactivity-guided fractionation (Figure S2). The MeOH layer was isolated by silica gel column chromatography (63–200 μM, 8.5 × 35 cm) under a gradient elution of n-hexane:EtOAc:MeOH (1:0:0→0:0:1) to yield 14 fractions.

Fraction one (FM-1, 243.8 mg) was loaded on a silica gel column (40–63 μM, 2.5 × 30 cm), and eluted with gradient mixtures of n-hexane:CHCl_3_ (1:0→5:1→1:1) to yield 11 fractions. A mixture of β-sitosterol and stigmasterol (**23** and **24**) (4.7 mg) was separated from FM-1-10 (16.1 mg) by preparative TLC (CHCl_3_:EtOAc, 25:1).

Fraction two (FM-2, 546.9 mg) was loaded on a silica gel column (40–63 μM, 3.0 × 33 cm) eluted with gradient solvent mixtures of n-hexane:CHCl_3_:MeOH (3:1:0→0:1:0→0:30:1→0:20:1→0:10:1), followed by purification using reversed phase solid–phase extraction (RP-SPE, C18 gel) to yield six fractions and fleminone (**16**) (20.6 mg). FM-2-3-SP2 (10.8 mg) was selected to be purified on RP-HPLC (MeOH:H_2_O, 3:1) yielding flemiflavanone A (**20**) (5.0 mg).

Fraction three (FM-3, 397.0 mg) was loaded on a silica gel column (40–63 μM, 3.0 × 32 cm) eluted with gradient solvent mixtures of CHCl_3_:EtOAc (1:0→20:1→15:1) to yield seven fractions. FM-3-4 (104.6 mg) was purified by RP–HPLC (MeOH:H_2_O, 7:3) to yield flemiphilippinin F (**3**) (15.9 mg) and isoderrone (**15**) (3.1 mg) and also two subfractions (FM-3-4-L1 and FM-3-4-L2) were obtained. Subfraction FM-3-4-L2 (10.9 mg) was purified by RP-HPLC (MeCN:H_2_O, 7:3) to yield 7-(3,3-dimethylallyl)genistein (**9**) (6.4 mg).

Fraction six (FM–6, 1.5 g) was chromatographed on a silica gel column (40–63 μM, 2 × 29 cm), which was eluted with CHCl_3_:EtOAc (8:1) to yield five fractions (FM-6-1 to FM-6-5). FM-6-1 (105.3 mg) was loaded on a silica gel column eluted with CHCl_3_:MeOH (30:1) to yield prunetin (**10**) (2.5 mg) and olmelin (**11**) (1.0 mg). FM-6-3 (308.1 mg) was selected for a silica gel column chromatography to yield three fractions. FM-6-3-1 (214.7 mg) was purified by RP-SPE with MeOH:H_2_O (3:2→1:0) to yield six fractions. FM-6-3-1-SP3 (11.2 mg) was purified by RP-HPLC (MeOH^−^:H_2_O, 4:1) to yield flemichin E (**2**) (1.5 mg). FM-6-3-1-SP5 (10.4 mg) was purified by RP-HPLC (MeOH:H_2_O, 4:1) to yield a mixture of **17** and **18** (2.5:1) (5.5 mg). FM-6-3-2 was purified by RP-SPE with MeOH:H_2_O (3:2→1:0) to yield six fractions. FM-6-3-2-SP3 (74.0 mg) was similarly purified by RP-HPLC (Waters, C18, 150 × 4.6 mm; MeOH:H_2_O = 3:1, HPLC–Shimadzu LC-10AT; UV-vis 254 nm; flow rate: 2 mL/min) to yield flemingin (**1**) (3.6 mg), **8** (48.0 mg) and **13** (1.2 mg). FM-6-5 (259.4 mg) was chromatographed on silica gel column chromatography, eluted with CHCl_3_:MeOH (30:1) to yield four fractions (FM-6-5-1 to FM-6-5-4). From FM-6-5-3, compound **21** (74.1 mg) was separated. FM-6-5-2 (110.9 mg) was isolated using the same solvent system (CHCl_3_:MeOH, 30:1) to yield **21** (92.3 mg).

Fraction seven (FM-7, 7.2 g) was recrystallized from n-hexane–EtOAc to render genistein (**5**) (898.0 mg). The remaining material of FM-7 was then separated by silica gel column chromatography and eluted with CHCl_3_-MeOH (25:1→20:1) to yield seven fractions (FM-7-1 to FM-7-7). From FM-7-6, compound **6** (2′-hydroxygenistein, 241.4 mg) was separated. FM-7-2 (1.1 g) was chromatographed on silica gel column (CHCl_3_–MeOH, 35:1) and purified by RP-SPE (MeOH:H_2_O, 40:1→1:0) and RP-HPLC (MeOH:H_2_O, 7:3) to yield cajanin (**7**) (11.3 mg), neoraufurane (**14**) (1.7 mg), erythrinin B (**12**) (3.2 mg), and flemiphilippinin D (**19**) (4.0 mg).

Fraction nine (FM–9, 5.2 g) was loaded on a silica gel column (63–200 μM, 4.5 × 21 cm) eluted with CHCl_3_:MeOH (6:1) to yield eight fractions. FM-9-7 (3.1 g) and FM-9-8 (583.4 mg) were combined and chromatographed on silica gel column eluted with gradient solvent mixtures of CHCl_3_:MeOH (10:1→6:1→5:1) to yield 4′-*O*-methyl-gallocatechin (**22**) (1.7 g). The isolated compounds were identified by NMR and the spectra were compared with the previously published data.

Flemingin (**1**), 5,2′,4′-trihydroxy-7,8-(1,2,2-trimethyl-dihydrofurano)isoflavone: yellow amorphous solid; ${\left[\alpha \right]}_{\text{D}}^{26}$: −13.45° (*c* 0.4, CHCl_3_); UV *λ*_max_ (MeOH) nm (log *ɛ*): 263 (4.41), 295 (3.99), 335 (4.52); IR (neat) V_max_ cm^−1^: 3377, 1651, 1615, 1506; HRESIMS *m*/*z* 377.1001 [M + Na]^+^ (calculated for C_20_H_18_O_6_: 377.1005); ^1^H and ^13^C NMR data: Table 1.

Flemichin E (**2**), (2*S*)-(5,2′-dihydroxy-6,7-(1,1-dimethylpyrano)-8-(3-methylbut-2-enyl)-4′,5′-(2- hydroxy-1,1-dimethylpyrano)flavanone: yellow amorphous solid; UV *λ*_max_ (MeOH) nm (log *ɛ*): 266 (4.63), 273 (4.65), 292 (4.30), 311 (4.18), 364 (3.65), 335 (4.52); IR(neat) V_max_ cm^−1^: 3366, 1642, 1601, 1507; HRESIMS *m*/*z* 529.2202 [M+Na]^+^ (calculated for C_30_H_34_O_7_: 529.2199).

### 3.4. Transgenic Plant Material and Estrogen–Like Reporter Assay

The *Arabidopsis* pER8:GUS line, with an estrogen receptor-based transactivator XVE (pER8) system, was developed originally by Brand *et al.* [7]. Seeds of pER8:GUS were grown on medium (1/2MS, 1% sucrose, 0.8% phytoagar), incubated at 4 °C for 24–36 h in dark conditions for vernalization, and then germinated under light for three days [43]. The germinated plants were then transferred into 24-well microtiter plates in the presence or absence of test samples and incubated at 24 °C for 48 h. Plants cultured with 0.31–10 nM 17β-estradiol were taken as the positive control.

### 3.5. Histochemical Assay

After incubation in the presence or absence of test samples, the plants were soaked in 0.2 mL per well of the GUS assay solution [50 mM Na_3_PO_4_ buffer (pH 7.0), 10 mM EDTA (pH 8.0), 2 mM X-Gluc, 0.5 mM K_3_Fe(CN)_6_, 0.5 mM K_4_Fe(CN)_6_, and 0.1% Triton X-100] in 24-well plates and were kept overnight at 37 °C. The plants were then soaked in 70% aqueous EtOH for 1 h to remove chlorophyll [44]. A ZEISS Axiovert 200 inverted microscope was used to examine GUS staining and to capture images with a digital camera. The minimum active concentration (MAC) of each sample was recorded upon the disappearance of the insoluble blue dye (5,5′-dibromo-4,4′-dichloro-indigo).

### 3.6. Reporter Gene Assay

Human breast adenocarcinoma cells MCF-7 obtained from the Bioresource Collection and Research Center (BCRC) were cultured in Eagle’s phenol-red free minimum essential medium (MEM) supplemented with dextran-charcoal treated serum, 2 mM l-glutamine and 10% fetal bovine serum (Gibco), penicillin, and streptomycin, in an atmosphere of 5% CO_2_ at 37 °C. Transfections were made using a liposome-based method (Lipofectamine 2000, Invitrogen Co., Carlsbad, CA, USA), according to the manufacturer’s instructions. Briefly, 0.2 μg of pERE-TA-SEAP plasmid (Clontech Laboratories, Inc., Mountain View, CA, USA) was transfected into 2 × 10^3^ cells in 100 μL of the growth medium per well for 6 h. Cells were washed and treated with samples of interest in growth medium for 48 h. Aliquots of culture media were analyzed for secreted alkaline phosphatase activity using the reporter Chemiluminescence Assay Kit, Phospha–LightTM (Applied Biosystems, Foster City, CA, USA). The MTT colorimetric assay was performed on the cells for assessing their corresponding cytotoxicity [45,46]. Finally, the estrogenic and antiestrogenic data were determined by the formula (final SEAP% = (SEAP/cell viability × 100) to avoid false data result from cytotoxicity [8]. Tests were carried out in triplicate or quadruplicate.

### 3.7. Human Neutrophil Superoxide Anion Generation and Elastase Release

Human neutrophils were obtained by means of dextran sedimentation and Ficoll centrifugation. Superoxide generation was carried out according to the procedures described previously [47,48]. Elastase release experiment was performed using MeO-Suc-Ala-Ala-ProValp-nitroanilide as the elastase substrate. Results of the superoxide anion production were collected by monitoring the superoxide dismutase–inhibitable reduction of ferricytochrome *c*.

### 3.8. Cytotoxicity

The following human cancer cell lines: liver (Hep3B and Hep G2), lung (A549), oral (Ca 9-22), and breast (MCF-7 and MDA-MB-231) cancer cell lines were obtained from the American Type Culture Collection. Cell viability was measured by the MTT colorimetric method [49]. Doxorubicin was used as a positive control. Absorbance at 550 nm was measured using a Multiskan Ascent microplate reader (Thermo Labsystems, Waltham, MA, USA). The mean IC_50_ is expressed as the concentration of the agent that reduced cell growth by 50% under the experimental conditions. The results represent the mean calculated from at least three independent experiments.

## 4. Conclusions

One new isoflavone (**1**) with unusual 2′,4′-dihydroxy substitutions and 23 known compounds were isolated from *F. macrophylla*. In the current study, the 1D, 2D NMR spectra and the stereochemical configuration of flemichin E (**2**) was reported for the first time. The results revealed that *F. macrophylla* major components exhibited potent binding affinity to ER, and were involved in ERE transcriptional activity acting as partial agonist and antagonists. The data collected from both reporter assays were in alignment with each other, suggesting that the transgenic plant system (pER8:GUS) or human breast cancer ER-positive MCF-7 cells can be used for screening new phytoestrogenic sources.

## Supplementary Information



## Figures and Tables

**Table 1:** ^13^C, ^1^H and HMBC data for fleminigin (**1**).

Position	δ_C_ (mult.) a	δ_H_ (mult., *J* in Hz) b	HMBC (^1^H→^13^C)
2	155.1	8.33 (s)	3, 4, 9, 1′
3	121.7		
4	181.9		
5	165.2		
6	94.7	6.53 (s)	5, 7, 8, 10
7	164.0		
8	112.8		
9	153.5		
10	106.8		
1′	110.0		
2′	158.3		
3′	104.4	7.08 (d, 2.4)	2′, 4′, 1′, 5′
4′	160.7		
5′	107.8	6.94 (dd, 8.4, 2.4)	1′, 3′
6′	133.4	7.62 (d, 8.4)	3, 2′, 4′
1″	90.9	4.43 (q, 6.4)	4″, 5″
2″	43.8		
3″	14.2	1.28 (d, 6.4)	1″, 2″, 4″, 5″
4″	25.5	1.37 (s)	8, 1″, 2″, 5″
5″	21.3	1.14 (s)	8, 1″, 2″, 3″, 4″

a ^13^C NMR (acetone*-d*_6_, 100 MHz) for **1**;

b ^1^H NMR (acetone-*d*_6_, 400 MHz) for **1**.

**Table 2:** ^13^C, ^1^H and HMBC data for flemichin E (**2**).

Position	δ_C_ (mult.) a	δ_H_ (mult., *J* in Hz) b	HMBC (^1^H→^13^C)
2	77.2	5.52 (dd, 13.2, 3.0)	
3	42.0	3.13 (dd, 17.1, 3.0)	2, 4
		2.82 (dd, 17.1, 13.2)	4
4	196.5		
5	156.7		
5-OH		12.9 (s)	5, 10
6	103.2		
7	158.7		
8	108.7		
9	159.7		
10	102.6		
1′	117.5		
2′	153.6		
3′	105.3	6.39 (s)	1′, 4′, 5′
4′	153.9		
5′	111.0		
6′	128.3	6.94 (s)	2, 4′, 3‴′
1″	115.6	6.64 (d, 10.2)	7, 3″
2″	126.2	5.51 (d, 10.2)	6, 3″
3″	78.2		
4″-CH_3_	28.3	1.43 (s)	2″, 3″, 5″
5″-CH_3_	28.4	1.45 (s)	2″, 3″, 4″

1‴	21.4	3.20 (2H, d, 7.8)	7, 8, 9, 2‴, 3‴
2‴	122.3	5.10 (td, 7.8, 1.8)	4‴, 5‴
3‴	131.7		
4‴-CH_3_	25.8	1.67 (d, 1.8)	2‴, 3‴, 5‴
5‴-CH_3_	17.8	1.68 (s)	2‴, 3‴, 4‴

1‴′	77.1		
2‴′	69.7	3.80 (dd, 5.4, 4.8)	
3‴′	30.7	3.01 (dd,16.8, 4.8)	4′, 5′, 6′, 2‴′
		2.71 (dd,16.8, 5.4)	4′, 5′, 6′, 1‴′, 2‴′
4‴′-CH_3_	22.2	1.37 (s)	1‴′, 2‴′, 5‴′
5‴′-CH_3_	24.8	1.33 (s)	1‴′, 2‴′, 4‴′

a ^13^C NMR (chloroform*-d*^1^, 150 MHz) for **2**;

b ^1^H NMR (chloroform*-d*^1^, 600 MHz) for **2**.

**Table 3:** Estrogenic activity of *F. macrophylla* isolated compounds.

Compounds	MAC (μM)
17β-estradiol	5.93 × 10^−4^
**1**	35.31
**3**	36.98
**4**	0.0139
**5**	0.0037
**6**	0.042
**7**	2.6
**8**	36.98
**12**	59.17
**13**	28.25
**16**	>500
**19**	58.96
**21**	118.48
**22**	312.50

**Table 4:** Anti-inflammatory activity of pure compounds isolated from *F. macrophylla*.

Compounds	IC_50_ (g/mL) or Inh%	

	Superoxide anion	Elastase release
**3**	3.74 ± 0.30 a,***	23.31 ± 3.82 c,***
**4**	6.82 ± 1.41 **	11.9 ± 5.02
**5**	0.66 ± 0.06 a	4.25 ± 1.23 a
**6**	1.89 ± 0.05 a	4.32 ± 0.14 a
**7**	8.86 ± 1.34 a	4.22 ± 0.72 a
**8**	2.18 ± 0.24 a	10.31 ± 3.82 b,***
**16**	10.31 ± 3.82	40.92 ± 11.08 b
**19**	73.08 ± 4.89 **	55.26 ± 5.94 c,**
**21**	ND	92.87 ± 2.27 b,**
**22**	29.37 ± 4.42 **	−4.34 ± 5.95

ND: not determined. Percentage of inhibition (Inh%) at 10 μg/mL. Results are presented as the mean ± S.E.M. (*n* = 3–4).

* *p* < 0.05,

** *p* < 0.01,

*** *p* < 0.001 compared with the control value.

a Concentration necessary for 50% inhibition (IC_50_).

b These compounds alone enhanced superoxide generation or elastase release by human neutrophils in the absence of formyl-methionyl-leucyl-phenylalanine (fMLP) and cytochalasin B (CB).

c This compound could induce superoxide generation or elastase release by human neutrophils in the presence of CB.
