# Supplementary material for: Phyto-SERM Constitutes from Flemingia macrophylla
Source: Int J Mol Sci. 2013 Jul 26;14(8):15578–94. doi: 10.3390/ijms140815578 (PMC3759874; doi:10.3390/ijms140815578)
Supplement: Supplementary file 1 [file ijms-14-15578-s001.pdf]

Supplementary Information

Figure S1. Bioactivity-guided isolation of *Flemingia macrophylla* (partition).

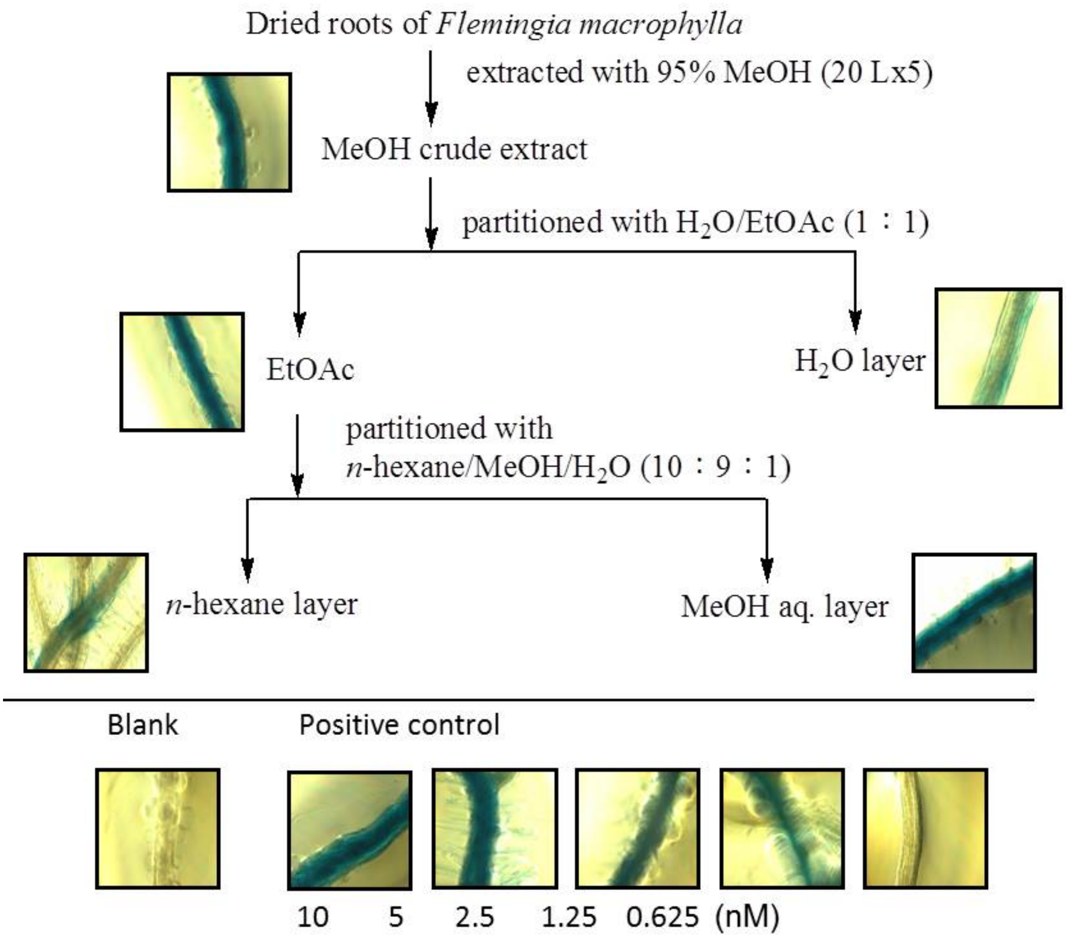

Figure S2. Bioactivity-guided isolation of *Flemingia macrophylla.* The transgenic plant assay system was used to determine estrogenic activity of subfractions which were isolated from MeOH layer.

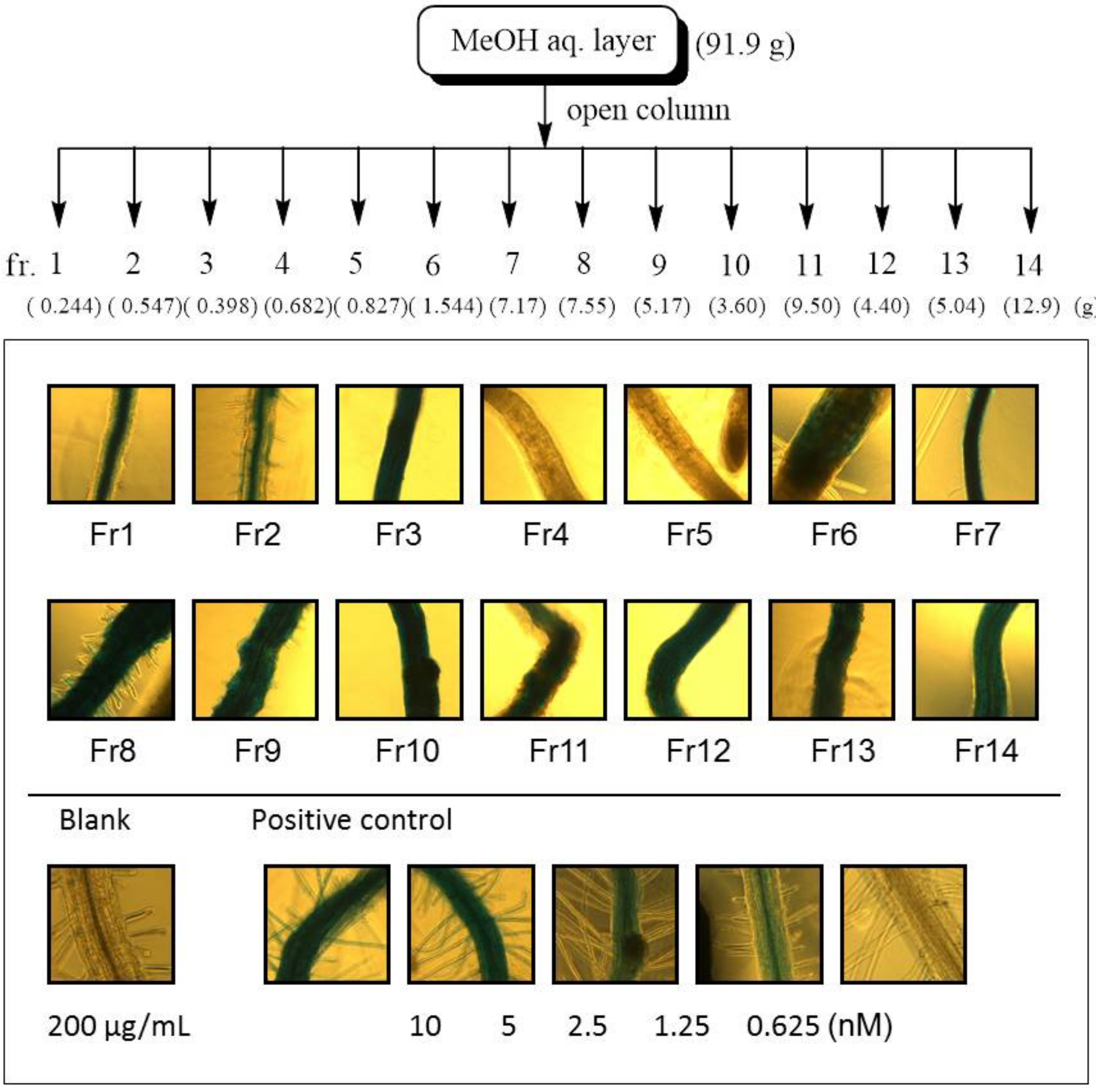

© 2013 by the authors; licensee MDPI, Basel, Switzerland. This article is an open access article distributed under the terms and conditions of the Creative Commons Attribution license (http://creativecommons.org/licenses/by/3.0/).
